# Absence of *kelch13* artemisinin resistance markers but strong selection for lumefantrine-tolerance molecular markers following 18 years of artemisinin-based combination therapy use in Mpumalanga Province, South Africa (2001–2018)

**DOI:** 10.1186/s12936-019-2911-y

**Published:** 2019-08-22

**Authors:** Jaishree Raman, Frank M. Kagoro, Aaron Mabuza, Gillian Malatje, Anthony Reid, John Frean, Karen I. Barnes

**Affiliations:** 1 Centre for Emerging Zoonotic and Parasitic Diseases, National Institute for Communicable Diseases, A Division of the National Health Laboratory Service, Sandringham, Johannesburg, Gauteng South Africa; 2 Wits Research Institute for Malaria, Faculty of Health Sciences, University of Witwatersrand, Johannesburg, South Africa; 3 UP Institute for Sustainable Malaria Control, Faculty of Health Sciences, University of Pretoria, Pretoria, South Africa; 4 Division of Clinical Pharmacology, Department of Medicine, University of Cape Town, Cape Town, South Africa; 5 Mahidol-Oxford Tropical Medicine Research Unit, Bangkok, Thailand; 6 Mpumalanga Provincial Malaria Elimination Programme, Nelspruit, Mpumalanga South Africa; 7 Operational Research Unit, Médecins Sans Frontières, Operational Centre, Brussels, Luxembourg

**Keywords:** Malaria, *Plasmodium falciparum*, Mutations, Mpumalanga Province, South Africa, ACT, *dhfr*, *dhps*, *crt*76, *mdr*86, *kelch13*, Resistance

## Abstract

**Background:**

The ability of *Plasmodium falciparum* parasites to develop resistance to widely used anti-malarials threatens malaria control and elimination efforts. Regular drug efficacy monitoring is essential for ensuring effective treatment policies. In low transmission settings where therapeutic efficacy studies are often not feasible, routine surveillance for molecular markers associated with anti-malarial resistance provides an alternative for the early detection of emerging resistance. Such a longitudinal survey of changes in the prevalence of selected molecular markers of resistance was conducted in the malaria-endemic regions of Mpumalanga Province, South Africa, where malaria elimination at a district-level is being pursued.

**Methods:**

Molecular analyses to determine the prevalence of alleles associated with resistance to lumefantrine (*mdr*86N, *crt*76K and *mdr1* copy number variation) and sulfadoxine–pyrimethamine (*dhfr* triple, *dhps* double, SP quintuple) were conducted between 2001 and 2018, while artemisinin resistance markers (*kelch13* mutations) were assessed only in 2018.

**Results:**

Parasite DNA was successfully amplified from 1667/2393 (70%) of malaria-positive rapid diagnostic tests routinely collected at primary health care facilities. No artemisinin resistance-associated *kelch13* mutations nor amplification of the *mdr1* gene copy number associated with lumefantrine resistance were observed. However, prevalence of both the *mdr*86N and *crt*76K alleles increased markedly over the study period, with all isolates collected in 2018 carrying these markers. SP quintuple mutation prevalence increased steadily from 14% in 2001 to 96% in 2018. Mixed alleles at any of the codons assessed were rare by 2018.

**Conclusion:**

No *kelch13* mutations confirmed or suspected to be associated with artemisinin resistance were identified in 2018. Although parasites carrying the *mdr*86N and *crt*76K alleles associated with reduced lumefantrine susceptibility were strongly selected for over the study period, nearing fixation by 2018, the marker for lumefantrine resistance, namely increased *mdr1* copy number, was not observed in this study. The increase in *mdr*86N and *crt*76K allele prevalence together with intense regional artemether–lumefantrine drug pressure, raises concern regarding the sustained artemether–lumefantrine efficacy. Regular, rigorous anti-malarial resistance marker surveillance across all three South African malaria-endemic provinces to inform case management is recommended.

## Background

**Fig. 1:**
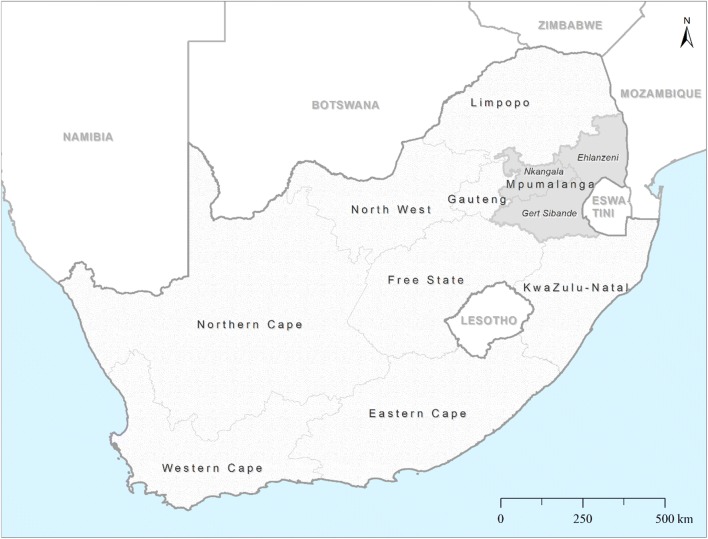
Map of South Africa showing the three endemic provinces and three municipal districts in Mpumalanga Province (Source: Collaborating Centre for Optimising Anti-malarial Therapy)

Although the global malaria burden has declined markedly since 2000, the disease remains a major cause of morbidity and mortality in Africa. In 2017, Africa accounted for 92% of the estimated 219 million malaria cases and 93% of all malaria deaths [1]. One of the major obstacles to effective malaria control and elimination remains the emergence and spread of anti-malarial drug resistance [2]. To increase anti-malarial efficacy and delay resistance, the World Health Organization (WHO) recommended artemisinin-based combination therapy (ACT) as first-line treatment for uncomplicated malaria [3]. South Africa was the first African country to deploy an ACT as first line in 2001 [4]. Artemether–lumefantrine replaced the failing sulfadoxine–pyrimethamine combination (SP) in KwaZulu-Natal (Fig. 1), one of South Africa’s three malaria-endemic provinces, in 2001 [4], with ACT introduced in the remaining two malaria-endemic provinces, Mpumalanga and Limpopo (Fig. 1) in 2003 and 2004, respectively [5]. By 2010, all sub-Saharan malaria-endemic African countries had adopted ACT [2].

Studies have since shown that ACT does not prevent the selection for molecular markers associated with resistance to the partner drugs, particularly if resistance to a partner drug had previously been described in the region [6, 7]. Even more concerning has been the confirmation of artemisinin-resistant parasites along the Thai-Cambodia border [8], the historic epicentre of anti-malarial drug resistance. Despite containment efforts, artemisinin-resistant parasites have spread rapidly across at least six countries in the Greater Mekong sub-region [9–11], with artemisinin resistance most recently also reported in eastern India [12]. While there have been isolated reports of artemisinin-resistant parasites from sub-Saharan Africa [13–15], artemisinin-resistant parasites have not yet become established on the continent [16], where their emergence and spread would severely threaten Africa’s malaria control efforts. Following over a decade of impressive gains in controlling malaria and advancing malaria elimination across southern Africa, the region has experienced malaria outbreaks during the last three malaria-transmission seasons [17]. This raised concerns that anti-malarial resistance may be contributing to the sharp increases in malaria case numbers, as had been observed previously with both chloroquine (CQ) and SP resistance [18].

To ensure efficacious ACT is in place, it is imperative that regular, rigorous anti-malarial drug efficacy/resistance monitoring occurs. The gold standard for assessing drug efficacy, in vivo therapeutic efficacy studies, are resource-intensive, and require a minimum of 50 patients [3]. This is often not feasible in low-transmission settings where few malaria cases are seen at each health facility, and most malaria occurs in highly mobile, migrant populations in whom follow-up for the required 28 to 42 days is challenging [19]. A more feasible, cost-effective method is assessing the prevalence of molecular markers associated with anti-malarial drug resistance and treatment failure [20]. Molecular markers associated with therapeutic efficacy of artemisinin, lumefantrine, SP, CQ, and amodiaquine (AQ) have been identified and validated [21–24].

Sustained implementation of effective interventions targeting both the malaria vector and parasite, following the 1999/2000 malaria epidemic, substantially reduced South Africa’s malaria burden, allowing the country to transition from malaria control (> 5 malaria cases/1000 population at risk) towards malaria elimination (< 1 malaria case/1000 population at risk) in 2012 [25]. As this low transmission intensity meant that adequately powered in vivo therapeutic efficacy studies were not feasible, the prevalence of molecular markers of anti-malarial resistance was used as a proxy for monitoring anti-malarial efficacy. This routine surveillance aimed to determine the prevalence and temporal changes of molecular markers associated artemisinin, lumefantrine and SP resistance in *Plasmodium falciparum* isolates extracted from malaria-positive rapid diagnostic tests (RDTs) obtained from primary health care (PHC) facilities in Mpumalanga Province (Fig. 1), South Africa (2001–2018), with a goal of ensuring that effective anti-malarial treatment policies are in place.

## Methods

### Country setting

Malaria in South Africa is currently restricted to the low-altitude border regions of three provinces: Limpopo, Mpumalanga and KwaZulu-Natal [26] (Fig. 1), with approximately 10% (4.9 million) of the country’s total population residing in malaria-risk areas [27]. The predominant malaria vector in South Africa is *Anopheles arabiensis* [28], with *P. falciparum* parasites the causative agent in most confirmed infections [28]. In line with South Africa’s guidelines for the treatment of malaria [29], all fever cases presenting at PHC facilities within a malaria-endemic district must be tested for malaria using a *P.* *falciparum*-specific RDT. Patients who are RDT malaria positive are treated in accordance with the guidelines [29].

### Study setting

**Fig. 2:**
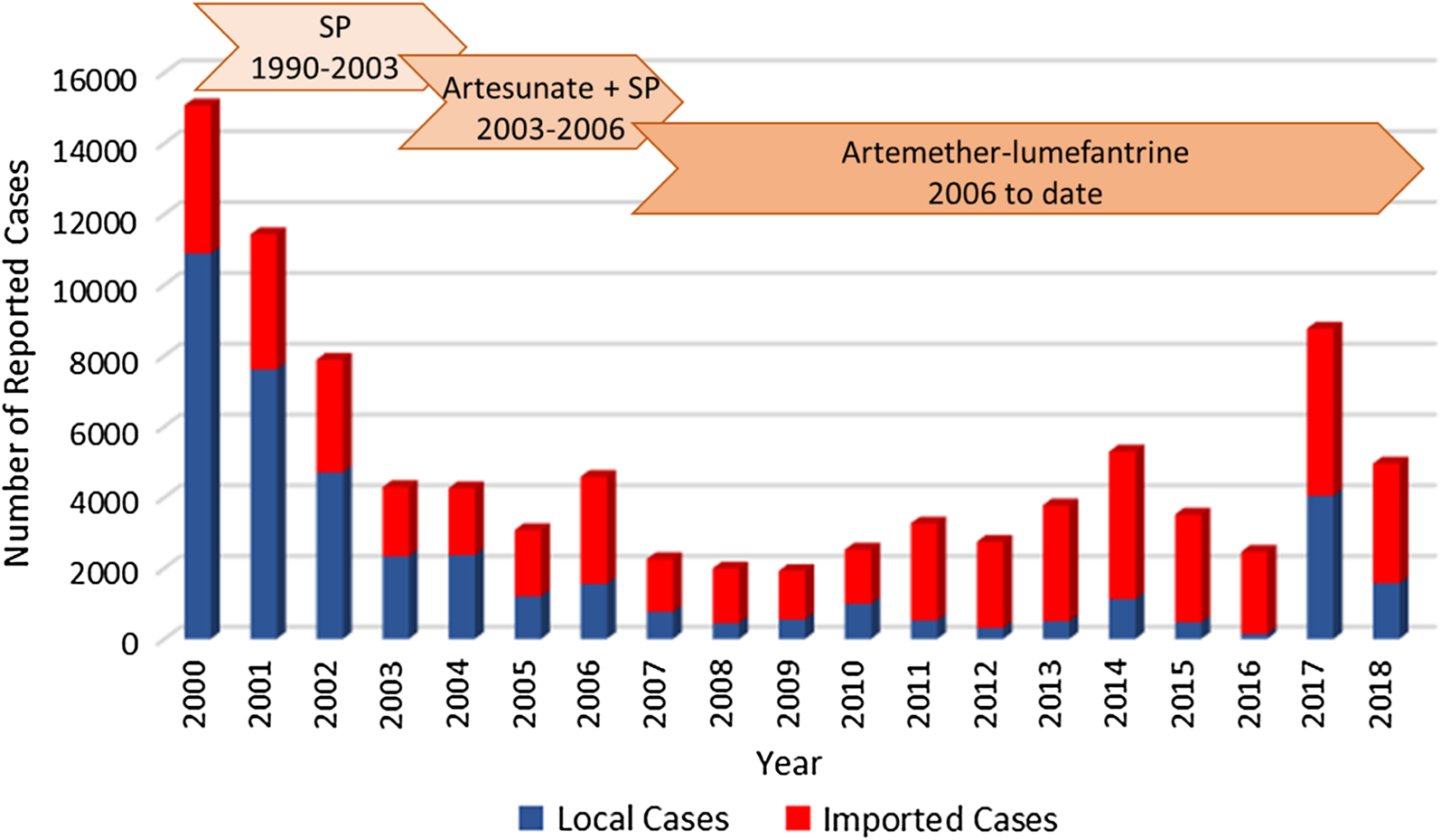
Number of local and imported cases reported in Mpumalanga Province by year with arrows indicating the first-line anti-malarial treatments deployed over the reporting period (2000–2018) (Source: South African National Department of Health)

Mpumalanga Province encompasses an area of 76,500 sq km with an approximate population of 4,040,000 [30]. The province comprises three districts (Fig. 1), with Ehlanzeni District (that shares a border with Mozambique and Eswatini) most affected by malaria [31]. Malaria transmission is seasonal but unstable, occurring during the rainy summer months from September to May, generally peaking in January and April coinciding with the peaks in people moving across the country’s border with Mozambique [32]. Despite sharp declines in locally acquired malaria cases, imported malaria case numbers continue to increase, accounting for 87% of the province’s reported cases by 2012 [32]. However, recent region-wide malaria epidemics reversed these gains, resulting in an increase in total case numbers and locally acquired infections [17], with the proportion of imported cases decreasing to 68% during the 2017/18 malaria season (Fig. 2).

SP replaced CQ as the drug of choice in Mpumalanga in 1997 [33], following a marked increase in CQ-treatment failures [34]. This was followed relatively soon by a sharp increase in markers associated with SP treatment failures, which was associated with increased gametocyte carriage, prompting the Mpumalanga Provincial Department of Health to implement an ACT policy in 2003, initially using artesunate plus SP, given cure rates above 90% with SP monotherapy [35, 36]. However, the continued selection for SP resistance markers following artesunate plus SP deployment in Mpumalanga [35, 36] and neighbouring southern Mozambique [7] supported the policy change to artemether–lumefantrine in 2006.

### Study design and data collection

The Malaria Molecular Laboratory of the South African Medical Research Council (SAMRC) partnered with the Mpumalanga Provincial Malaria Control Programme to conduct the anti-malarial resistance marker analysis using malaria-positive RDTs from PHC facilities, until the closure of the Malaria Laboratory in 2013 as part of the SAMRC restructuring. The surveillance programme was revived by the Laboratory for Antimalarial Resistance Monitoring and Malaria Operational Research of the National Institute for Communicable Diseases (NICD) and the Mpumalanga Provincial Malaria Elimination Programme during the 2017/2018 malaria season. This cross-sectional, anti-malarial resistance marker prevalence study was conducted between 2001 and 2018 using malaria-positive RDTs collected from various PHC facilities within the malaria-endemic districts of Mpumalanga Province. The collected malaria-positive RDTs were transported to the SAMRC on an ad hoc basis but were couriered weekly to the NICD.

### Molecular analysis

In the laboratory, parasite DNA was extracted from the positive RDTs (ICT™, Global Diagnostics, Cape Town, South Africa; SD Bioline, SD, Korea; First Response, Premier Technologies, India) using a modified Chelex method [37] from 2001 until 2011 and the Qiagen DNA mini extraction kit (Qiagen, Germany) in 2018. Once confirmed as *P. falciparum* positive by either qPCR [38] or multiplex PCR [39], polymorphism analysis of *dhfr*, *dhps*, *crt*, and *mdr1* genes was conducted. Molecular markers associated with SP resistance were assessed in all study years using all available DNA isolates. Budget constraints limited the analysis of lumefantrine tolerance/resistance markers in the *mdr1* (*mdr*N86Y and *mdr1* copy number variations) and *crt* (*crt*K76T) genes, to 2001, 2011 and 2018, with an additional assessment of the *mdr1* markers conducted in 2009 (Table 1). As the *kelch13* markers associated with artemisinin resistance were identified in 2014, these were only assayed in the samples collected in 2018.

**Table 1:** Number of parasite isolates analysed by year and mutation marker in Mpumalanga Province, South Africa (2001–2018)

Year	Number of RDTs collected	DNA successfully extracted (%)	Number of parasite isolates analysed				
			SP resistance markers^a^ (%)	Lumefantrine tolerance/resistance markers			Artemisinin resistance markers^e^ (%)
				*mdr*N86Y^b^ (%)	*mdr1* copy number^c^ (%)	*crt*K76T^d^ (%)	
2001	195	93 (48)	93 (100)	14 (15)	12 (13)	22 (24)	–
2008	190	57 (30)	57 (100)	–	–	–	–
2009	190	81 (42)	81 (100)	81 (100)	73 (90)	–	–
2010	95	58 (61)	58 (100)	–	–	–	–
2011	663	596 (90)	596 (100)	558 (94)	390 (65)	333 (56)	–
2012	97	97 (100)	97 (100)	–	–	–	–
2018	963	686 (71)	655 (96)	514 (75)	482 (70)	452 (66)	532 (78)
Total	2393	1667 (70)	1637 (98)	1167 (70)	957 (57)	807 (48)	532 (32)

*RDTs* rapid diagnostic tests, *DNA* deoxyribose nucleic acid, *SP* sulfadoxine–pyrimethamine

^a^Mutations at codons *dhfr*51, *dhfr*59, *dhfr*108, *dhfr*164 of the dihydrofolate reductase (*dhfr*) gene and *dhps*436, *dhps*437, *dhps*540 and *dhps*581 of the dihydropteroate synthetase (*dhps*) genes were assessed

^b^Mutations at codon *mdr*86 of the multidrug resistance 1 (*mdr1*) gene were assessed

^c^Variations in the *mdr1* gene copy number were assessed

^d^Mutations at codon *crt*76 of the chloroquine resistance transporter (*crt*) gene were assessed

^e^Mutations at 25 codons in the propeller domain of the *kelch13* gene were assessed

Primers, PCR amplification conditions and restriction endonucleases used to detect polymorphisms in the *dhfr* (codons 51, 59, 108, 164), *dhps* (codons 436, 437, 540 and 581), *mdr1* (codon 86), and *crt* (codon 76) genes have been described previously [6, 40, 41]. Digestion products were separated on a 2% agarose gel using electrophoresis, then visualized and photographed using either a MiniBIS™ (BioSystematica, UK) or Omega Fluor™ (Gel Company, USA) documentation system. Codons were classified as either wild-type, mutant or mixed (both mutant and wild-type genotypes present in an individual sample). Genotyping assays were run in duplicate, with a third assay performed on any discordant results. When calculating overall prevalence of infections with mutant genotypes, codons with mixed genotypes were grouped with pure mutant codons.

Copy number of the *mdr1* gene was assessed using the qPCR method, primers, probes, and qPCR cycling conditions previously described by Price et al. [42]. Every qPCR run contained three reference DNA samples from D10 and Fac8 clones, having an *mdr1* copy number of one and three, respectively, as well as a no-template control. Assays were repeated if the threshold cycle values were greater than 35.

The propeller domain of the *kelch13* gene was amplified using the protocol of Talundzic et al. [43]. The amplified products were sent to Inqaba Biotechnologies (Pretoria, South Africa) for Sanger sequencing. Sequences were then aligned against a reference *P. falciparum kelch13* gene (XM_001350122.1) using a BLAST search and BioEdit Software to detect polymorphisms after codon 400 of the *kelch13* gene, the genetic region containing the mutations associated with delayed parasite clearance in Southeast Asia [24, 44].

### Statistical analysis

Statistical analysis was performed using Stata 15.0 (Stata Corp., College Station, TX, USA). Univariate analysis was conducted to determine if year (proxy for time since change in antimalarial treatment policy) was significantly associated with mutation prevalence. Confidence limits (CI) were set at 95% with a p value < 0.05 considered to have statistical significance.

### Spatial data exploration and curation

A dataset of molecular markers with clinic names was imported for cleaning and analysis into R Studio version 3.5.2. Coordinates and location information was secondarily added by linking the molecular dataset with a facility and localities location dataset maintained at the NICD that contained facility coordinates.

Provincial malaria control programme information officers assisted with the identification of health facilities/localities data that did not match in the NICD facility database and provision of missing coordinates information. A few facility/locality observations (9%) lacked adequate information to allow for proper identification.

For verification of the coordinates, all the matched locations were further explored using Google Maps. Two locations that fell outside the study area were removed, resulting in a final dataset comprising 90 locations and 1658 (73%) observations from the molecular marker dataset.

### Spatial analysis

Using ArcMap 10.6.1, the molecular markers dataset was linked to the curated location coordinates to produce the spatial dataset. All country and sub-level shapefiles were obtained from an open-source platform of the latest Database of Global Administrative Areas (GADM version 3.6 released on 6 May 2018) [45]. All coordinates were assumed to have been based on the WGS 1984 coordinate system, and the Esri Display XY dialogue was used to integrate longitudes and latitudes of the localities on the maps.

Four important themes in defining molecular markers dictated the choices of colours and legend, namely tolerant, mixed, sensitive or being absent. Colour-friendly choices were picked from the colour brewer’s toolkit [46]. Graduated symbols of equal proportions were also used throughout the maps for denoting the sample size of the markers involved for each locality to enhance interpretability [47].

### Ethics approval

Approval for this study was obtained from the Mpumalanga Provincial Department of Health (MP_2015RP53_229), and the University of Witwatersrand Human Research Ethics Committee: Medical (M160229). It also met the criteria for studies of routinely collected data of the Ethics Review Board of *Médecins Sans Frontières*.

## Results

### *Plasmodium* DNA isolates

The number of malaria-positive RDTs submitted for analysis increased over the study period, from under 200 per year between 2001 and 2010, to 663 in 2011 and 963 in 2018 (Table 1). Overall, parasite DNA was successfully extracted and amplified from 70% (1667/2393) of the malaria-positive RDTs received for analysis. Between 2001 and 2009 DNA was successfully extracted from 40% of the RDTs received, increasing to 61% in 2010 and consistently over 70% between 2011 and 2018. Method of DNA extraction did not appear to influence the success of DNA extraction.

### Artemisinin resistance marker prevalence

**Fig. 3:**
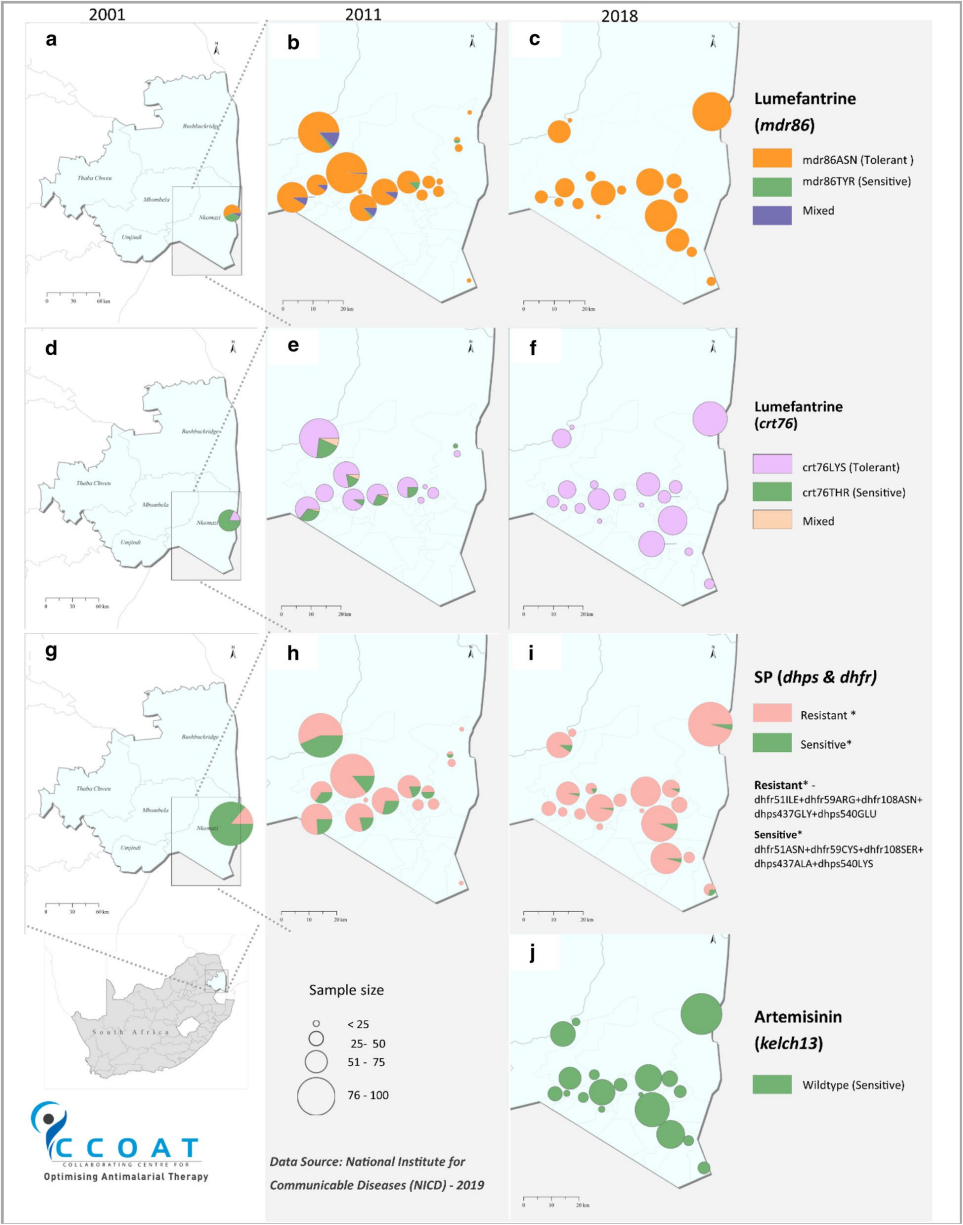
Spatial and temporal changes in the prevalence of **a**–**c** *mdr*86ASN lumefantrine tolerance marker, **d**–**f** *crt*76LYS lumefantrine tolerance marker, **g**–**i** the quintuple SP resistance marker and **k** the *kelch13* markers in Ehlanzeni District, Mpumalanga Province, South Africa (2001–2018)

Presence of the *kelch13* artemisinin resistance markers could be determined in 78% (532/686) of the samples from which parasite DNA was extracted in 2018. Not one of the 25 polymorphisms confirmed or suspected to be associated with delayed parasite clearance in Southeast Asia was detected in these samples (Fig. 3j).

### Lumefantrine tolerance marker prevalence

**Fig. 4:**
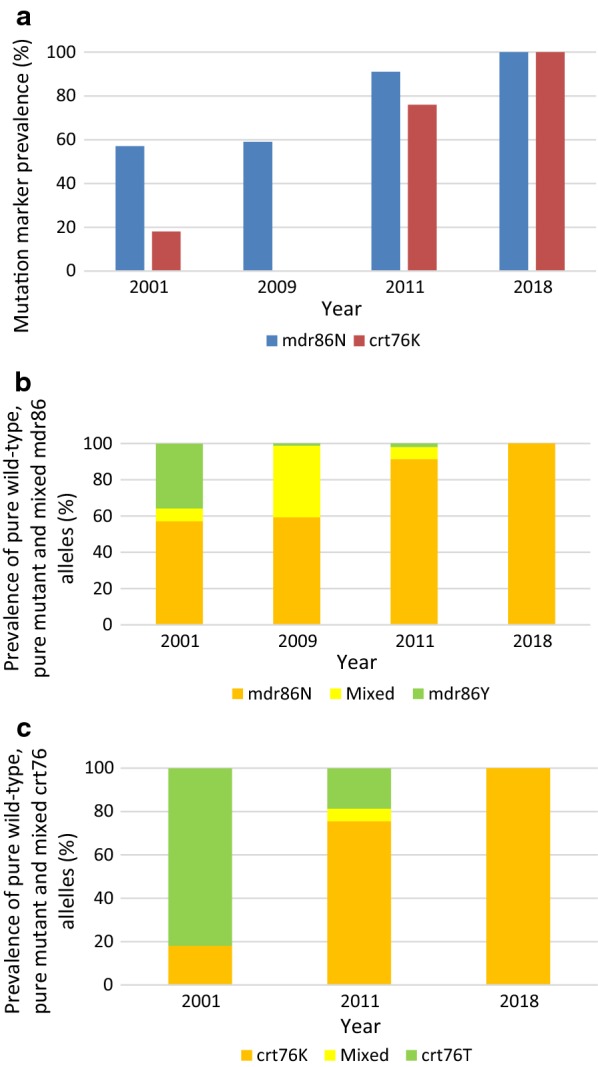
**a** Prevalence of the *mdr*86Y and *crt*76T mutations in *Plasmodium falciparum* isolates from Ehlanzeni District, Mpumalanga Province, South Africa, by year (2001–2018). Mutations at both codons were assessed in 2001, 2011 and 2018, with mutations in the *mdr1* gene also assessed in 2009, **b** changes in the prevalence of pure wild-type *mdr*86N, pure mutant *mdr*86Yand mixed *mdr*N86Y alleles (2001–2018) and **c** prevalence of pure wild-type *crt*76K, pure mutant *crt*76T and mixed *crt*K76T alleles (2001–2018)

Prevalence of the pure *mdr*86N wild-type allele (associated with lumefantrine tolerance but CQ and AQ sensitivity) increased significantly over the study period (p < 0.0001), from 57% (8/14) in 2001 to 59% (48/81) in 2009 and 91% in 2011, reaching fixation (100%, 514/514) by 2018 (Figs. 3a–c and 4a). Although the prevalence of the pure *mdr*86N wild-type allele was similar in 2001 and 2009, there was a sharp increase in mixed *mdr*N86Y alleles from 7% (1/14) to 39.5% (31/81) over this period (Fig. 4b). Thereafter the prevalence of the mixed *mdr*N86Y alleles decreased markedly, with no mixed alleles detected in the 2018 samples (Figs. 3c and 4b). No variation in *mdr1* gene copy number was observed in any sample analysed over the study period.

At baseline (2001), only 18% (4/22) of the samples analysed carried the *crt*76K wild-type allele (Figs. 3d and 4a) associated with lumefantrine tolerance. However, prevalence of this allele increased significantly to 75.7% (252/333) in 2011 (Figs. 3e and 4a) and reached fixation, being present on all 452 samples analysed in 2018 (p < 0.001; Figs. 3f and 4a). Mixed *crt*76 alleles were rare, only detected in 2011 (Figs. 3e, f and 4c). Over the study period isolates carrying the *crt*76K wild-type allele were over 10 times more likely to carry the *mdr*86N allele (OR: 10.67; 95% CI 5.5–20.7; p < 0.0001), with all 452 samples assayed for the *crt*76 mutation in 2018 carrying the wild-type *mdr*86 allele.

### SP resistance marker prevalence

**Fig. 5:**
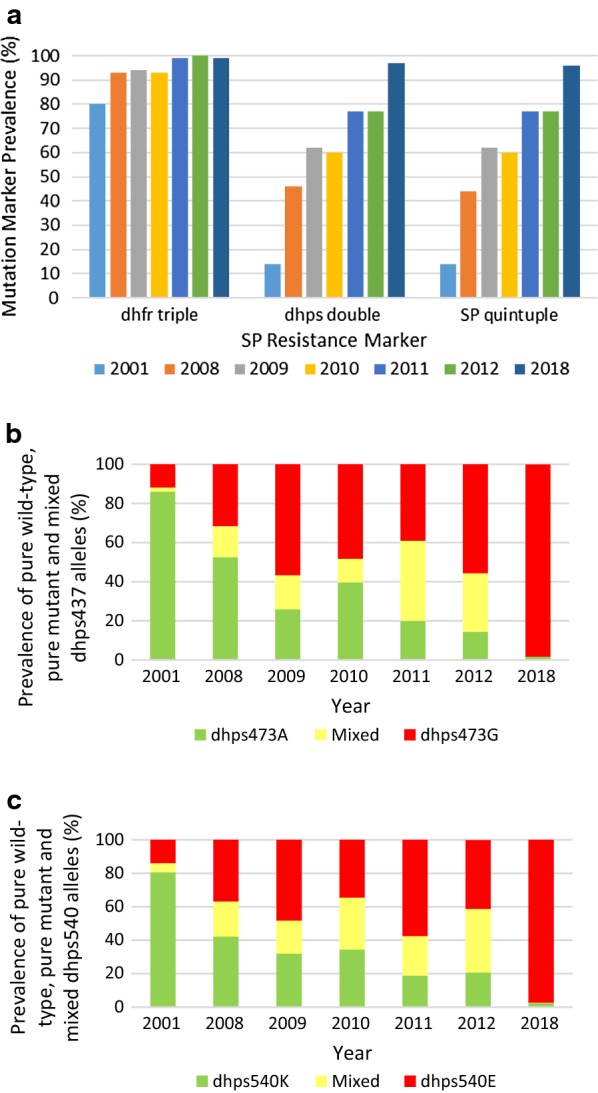
**a** Prevalence of *dhfr* triple, *dhps* double and SP quintuple mutations in *Plasmodium falciparum* isolates from Mpumalanga Province, South Africa, by year (2001–2018), **b** changes in the prevalence of pure wild *dhps*437A, mutant *dhps*437G and mixed *dhpsA*437G alleles (2001–2018) and **c** pure wild *dhps*540K, mutant *dhps*540E and mixed *dhps*K540E alleles (2001–2018)

The *dhfr* triple haplotype (codons *dhfr*51I, *dhfr*59R and *dhfr*108N) associated with pyrimethamine resistance increased significantly (p < 0.0001) over the study period, from 80% (74/92) in 2001 to 99% (653/658) by 2018 (Fig. 5a). This paved the way for parasites carrying the *dhps* double mutation to increase more steeply during the study (p < 0.001) from 14% (13/93) in 2001 to 97% (635/655) in 2018, which was mirrored by the SP quintuple mutation increasing from 14% (13/93) in 2001 to 96% (630/655) in 2018 (Figs. 3g–i, 5a); p-values < 0.001 for both. Mixed *dhps*437 and *dhps*540 alleles were seldom detected at the start of the study, with most isolates carrying the *dhps*437A and *dhps*540K wild-type alleles (Fig. 5b, c). Over the study period the prevalence of both the mixed, as well as mutant *dhps*437 and *dhps*540, alleles increased (Fig. 5b, c). Mixed *dhps*437 alleles peaked at 41% in 2011 but declined to 30% by 2012 (Fig. 5b). In contrast, mixed *dhps*540 alleles continued to increase over the study period, constituting 38% of all *dhps*540 alleles analysed in 2012 (Fig. 5c). However, by 2018 mixed alleles were extremely rare with over 97% of the samples analysed carrying pure mutant *dhps*437 and *dhps*540 alleles (Fig. 5b, c). Mutations at codons *dhfr*164 and *dhps*581 were not detected in any of the samples tested.

## Discussion

The rapid selection of malaria parasites resistant to first-line anti-malarials is of great concern to the affected communities, clinicians, malaria researchers, and malaria control specialists. Regular drug efficacy monitoring using therapeutic efficacy studies or molecular resistance marking has been recommended by the WHO to enable early detection of emerging resistance and facilitate prompt policy changes before therapeutic efficacy falls below 90% [2]. Data presented here describe the first long-term study in Mpumalanga Province, South Africa, assessing temporal trends in anti-malarial resistance marker prevalence.

Over the 18-year study period, parasites carrying the *mdr*86N and *crt*76K wild-type alleles associated with lumefantrine tolerance were strongly selected for, with all parasites analysed in 2018 carrying these wild-types alleles. Similar selection for lumefantrine tolerance has been observed across Africa, particularly where artemether–lumefantrine is first-line treatment [48]. Parasites with the *mdr*86N wild-type allele have been shown to be more likely to recrudesce after artemether–lumefantrine treatment compared to parasites with the *mdr*86Y mutant allele [49] and are more able to survive exposure to considerably higher lumefantrine concentrations if they also carry the *mdr*184F and *mdr*1246D alleles [50]. Despite the increased wild-type *mdr*86N allele prevalence, amplification of *mdr1* gene copy number, linked to artemether–lumefantrine treatment failures in Southeast Asia [42], was not observed in this study and is rare in Africa [48], suggesting an alternative mechanism may be associated with lumefantrine resistance in Africa. It is possible that the strong selection for *mdr*86N and *crt*76K wild-type alleles was driven by a reduction in CQ drug-pressure, as previously seen in Malawi [51]. However, as CQ has not been used in Mpumalanga since 1997 [33] and the significant increases in *mdr*86N and *crt*76K wild-type haplotypes were only observed after artemether–lumefantrine had been deployed in the province, the selection for these alleles in Mpumalanga is most likely driven by lumefantrine drug pressure.

In spite of the increased pressure on the artemisinin component, given reduced lumefantrine susceptibility and artemisinin-resistant parasites now being reported from India [12] as well as six other countries in Greater Mekong sub-region [10], artemisinin resistance has not yet been established in Africa. However, there have been reports of decreased artemether–lumefantrine efficacy from certain African countries [52, 53], raising concerns over the therapeutic longevity of artemether–lumefantrine, the most widely recommended ACT in Africa and first-line anti-malarial treatment in all southern African countries [1]. Artemether–lumefantrine therapeutic efficacy data from a multi-year, multi-centre study assessing the safety of single low-dose primaquine in Mpumalanga Province reported a 100% PCR-corrected adequate clinical and parasitological response [54]. However, the majority of the study participants were adult Mozambicans, who most likely had acquired some immunity to malaria due the higher transmission intensity in that country. It is plausible that this acquired immunity contributed in part to the high cure rate, highlighting the need for regular drug efficacy monitoring in South Africa and other low transmission countries, where acquired immunity in locally transmitted cases is unlikely.

Concurrently with selection for lumefantrine tolerance, was a strong selection for parasites carrying the SP quintuple mutation associated with SP treatment failure. Molecular resistance studies from South Africa [55], Mozambique [7, 20] and Malawi [56] have confirmed that ACT (artesunate plus SP and artemether–lumefantrine) deployment has not halted the selection of molecular markers associated with SP treatment failures. In Gaza Province, Mozambique, which borders Mpumalanga Province, SP quintuple mutation prevalence neared 80% in 2010 despite the ACT, artemether–lumefantrine, being first-line treatment in that country since 2008 [20]. A similar pattern was observed in Malawi, where almost all parasites analysed carried the SP quintuple mutation 5 years after SP had been replaced by an ACT as the anti-malarial of choice [55]. Possible reasons for the continued selection of SP resistance markers include sustained regional drug pressure due to the continued use of SP for intermittent preventive treatment (IPT) primarily in pregnancy in many southern African countries with higher intensity malaria transmission [1], and/or cross-resistance resulting from the widespread use of cotrimoxazole, an antifolate–sulfonamide drug combination similar to SP, as prophylaxis against opportunistic infections in people living with HIV/AIDS [57].

Strengthening of the malaria surveillance system in Mpumalanga Province since 2010 has positively impacted the quantity and more importantly, quality of the RDTs received for analysis. Regular refresher training on administration and interpretation of RDTs results, together with the implementation of guidelines for the packaging (packaged in zip-lock packets with desiccant) and transportation (routine scheduled submission) of used RDTs as part of this system strengthening has resulted in a significant increase in parasite DNA successfully extracted from the RDTs. Although other researchers in Africa have previously used RDTs as source of parasite DNA for anti-malarial resistance detection [58–60], this is one of the first studies to use RDTs from a programmatic and operational level for routine anti-malarial resistance marker surveillance. This study, therefore, re-enforces the usefulness of RDTs as a source of parasite DNA in resource-limited rural settings where collection and appropriate storage of blood samples may not be feasible.

Unfortunately, as the archived RDTs used in this study contained no patient identifiers it was not possible to link haplotype to a clinical outcome and/or patient characteristics, limiting the immediate clinical impact of the resistance marker data generated. This shortcoming is being addressed with the roll-out of the smart surveillance for malaria elimination initiative in Mpumalanga, where resistance data will be linked to anonymized patient data in almost real time. In line with the revised WHO surveillance guidelines [61], the provincial malaria control teams attempt to follow-up all notified malaria cases to ensure cure and drug compliance. However, the majority of malaria cases occur in the large mobile and migrant populations on the border with Mozambique. This, together with well over 1000 cases annually, precludes the integrated therapeutic efficacy studies recommended by the WHO [61] for use in low-transmission, pre-elimination settings.

Maps displaying the prevalence and spatial–temporal distribution for resistance markers will be regularly generated to help inform policy in the province. More importantly, containment efforts can be rapidly targeted at the individual and appropriate community level (based on residential and source location) should the first case of artemisinin resistance be identified in this part of southern Africa. To ensure South Africa is able to respond rapidly to any emerging anti-malarial resistance parasites, routine surveillance using RDTs should be expanded to the other two malaria-endemic provinces as a matter of urgency.

## Conclusion

This study highlights the feasibility and suitability of using RDTs as a source of parasite DNA for routine anti-malarial resistance surveillance particularly in rural, low-prevalence, resource-strained settings with malaria occurring mostly in mobile and migrant populations. The regionwide sustained deployment of artemether–lumefantrine has conferred a strong selective advantage to lumefantrine-tolerant parasites (carrying the wild-type *mdr*86N and *crt*76K alleles), enabling them to become the dominant parasite-type circulating within the southern African region. This rise in lumefantrine tolerance has increased the burden on the artemisinin component to clear the parasite load, which has the potential to increase the risk of artemisinin resistance and threaten the sustained efficacy of artemether–lumefantrine. Sustained, rigorous surveillance for molecular markers of anti-malarial resistance is recommended to allow for the early detection of resistance, informing treatment policy and facilitating prompt containment efforts should any case of artemisinin resistance be identified. This is essential, given the devastating impact both CQ and SP resistance have had historically in southern Africa, and the malaria epidemiological similarities between this region and the areas in the Greater Mekong sub-region where resistance to widely used anti-malarials, including artemisinins, first emerged.

## Data Availability

The datasets used and analysed during this study are available from the corresponding author on reasonable request.

## Abbreviations

ACT: artemisinin-based combination therapy
AQ: amodiaquine
*crt*76: codon 76 of the *P. falciparum* chloroquine resistance transporter gene
CQ: chloroquine
*dhfr*: dihydrofolate reductase
*dhfr*51: codon 51 of the dihydrofolate reductase gene
*dhfr*59: codon 59 of the dihydrofolate reductase gene
*dhfr*108: codon 108 of the dihydrofolate reductase gene
*dhfr* triple mutation: presence of mutations at *dhfr* codons 51, 59 and 58 of the dihydrofolate reductase gene
*dhps*: dihydropteroate synthetase
*dhps*436: codon 436 of the dihydropteroate synthetase gene
*dhps*437: codon 437 of the dihydropteroate synthetase gene
*dhps*540: codon 540 of the dihydropteroate synthetase gene
*dhps*581: codon 581 of the dihydropteroate synthetase gene
*dhps* double: presence of mutations at codons 437 and 540 of the dihydropteroate synthetase gene
DNA: deoxyribose nucleic acid
*mdr1*86: codon 86 of the *P. falciparum* multi-drug resistance gene 1
NICD: National Institute for Communicable Diseases
PCR: polymerase chain reaction
PHC: primary health care facilities
qPCR: quantitative polymerase chain reaction
RDT: rapid diagnostic test
SAMRC: South African Medical Research Council
SP: sulfadoxine–pyrimethamine
SP quintuple mutation: presence of both the *dhfr* triple and *dhps* double mutations
